# The Application of Genomics to Emerging Zoonotic Viral Diseases

**DOI:** 10.1371/journal.ppat.1000557

**Published:** 2009-10-26

**Authors:** Bart L. Haagmans, Arno C. Andeweg, Albert D. M. E. Osterhaus

**Affiliations:** Department of Virology, Erasmus Medical Center, Rotterdam, The Netherlands; The Scripps Research Institute, United States of America

## Abstract

Interspecies transmission of pathogens may result in the emergence of new infectious diseases in humans as well as in domestic and wild animals. Genomics tools such as high-throughput sequencing, mRNA expression profiling, and microarray-based analysis of single nucleotide polymorphisms are providing unprecedented ways to analyze the diversity of the genomes of emerging pathogens as well as the molecular basis of the host response to them. By comparing and contrasting the outcomes of an emerging infection with those of closely related pathogens in different but related host species, we can further delineate the various host pathways determining the outcome of zoonotic transmission and adaptation to the newly invaded species. The ultimate challenge is to link pathogen and host genomics data with biological outcomes of zoonotic transmission and to translate the integrated data into novel intervention strategies that eventually will allow the effective control of newly emerging infectious diseases.

## Emerging Zoonotic Viruses

Most of the well-known human viruses persist in the population for a relatively long time, and coevolution of the virus and its human host has resulted in an equilibrium characterized by coexistence, often in the absence of a measurable disease burden.

When pathogens cross a species barrier, however, the infection can be devastating, causing a high disease burden and mortality. In recent years, several outbreaks of infectious diseases in humans linked to such an initial zoonotic transmission (from animal to human host) have highlighted this problem. Factors related to our increasingly globalized society have contributed to the apparently increased transmission of pathogens from animals to humans over the past decades; these include changes in human factors such as increased mobility, demographic changes, and exploitation of the environment (for a review see Osterhaus [1] and Kuiken et al. [2]). Environmental factors also play a direct role, and many examples exist. The recently increased distribution of the arthropod (mosquito) vector *Aedes aegypti*, for example, has led to massive outbreaks of dengue fever in South America and Southeast Asia. Intense pig farming in areas where frugivorous bats are common is probably the direct cause of the introduction of Nipah virus into pig populations in Malaysia, with subsequent transmission to humans. Bats are an important reservoir for a plethora of zoonotic pathogens: two closely related paramyxoviruses—Hendra virus and Nipah virus—cause persistent infections in frugivorous bats and have spread to horses and pigs, respectively [3].

The similarity between human and nonhuman primates permits many viruses to cross the species barrier between different primate species. The introduction into humans of HIV-1 and HIV-2 (the lentiviruses that cause AIDS), as well as other primate viruses, such as monkeypox virus and Herpesvirus simiae, provide dramatic examples of this type of transmission. Other viruses, such as influenza A viruses and severe acute respiratory syndrome coronavirus (SARS-CoV), may need multiple genetic changes to adapt successfully to humans as a new host species; these changes might include differential receptor usage, enhanced replication, evasion of innate and adaptive host immune defenses, and/or increased efficiency of transmission. Understanding the complex interactions between the invading pathogen on the one hand and the new host on the other as they progress toward a new host–pathogen equilibrium is a major challenge that differs substantially for each successful interspecies transmission and subsequent spread of the virus.

## Genomics of Zoonotic Viruses and Their Hosts

New molecular techniques such as high-throughput sequencing, mRNA expression profiling, and array-based single nucleotide polymorphism (SNP) analysis provide ways to rapidly identify emerging pathogens (Nipah virus and SARS-CoV, for example) and to analyze the diversity of their genomes as well as the host responses against them. Essential to the process of identification and characterization of genome sequences is the exploitation of extensive databases that allow the alignment of viral genome sequences and the linkage of these genomics data to those obtained by classical viral culture and serological techniques, and epidemiological, clinical, and pathological studies [4]. Extensive genetic analysis of HIV-1, for example, has provided clues to the geography and time scale of the early diversification of HIV-1 strains when the virus emerged in humans. HIV-1 strains are divided into multiple clades, each of which has independently evolved from a simian immunodeficiency virus (SIV) that naturally infects chimpanzees in West and Central Africa. Current estimates date the common ancestor of HIV-1 to the beginning of the twentieth century [5].

Because zoonotic pathogens typically may cause variable clinical outcomes in human hosts that differ in age, nutritional status, genetic background, and immunological condition, deciphering the complex interactions between evolving pathogens and their hosts is a great challenge. The genome sequences of many host species have become available the last decade, and with them a range of novel tools are available to study virus–host interactions at the molecular level. This progress, together with advances in high-throughput sequencing technology and, not least, in (bio)informatics and statistics, allows us to analyze the “genome-wide” networks of gene interactions that control the host response to pathogens. By comparing and contrasting the outcomes of infection with closely related pathogens in different but related host species, we can further delineate the various host pathways involved in the different outcomes. The power of this approach was nicely demonstrated for SIV infection of various primate host species. Natural reservoir hosts of SIV do not develop AIDS upon infection, whereas non-natural hosts, such as rhesus macaques and pig-tailed macaques, when infected experimentally with SIV, develop AIDS in a similar manner to HIV-infected humans. Transcriptional profiling indicates that SIV infection of these species produces a distinctive host response [6]. SIV-infected primates with symptoms of AIDS have a high viral load, immune activation, and loss of certain types of T cells, whereas SIV-infected sooty mangabeys (the species from which HIV-2 is thought to have originated) have substantially lower levels of innate immune activation than the symptomatic primates, partly due to the production of less interferon-α by plasmacytoid dendritic cells in response to SIV and other Toll-like receptor ligands [7]. Identification of host factors that restrict HIV infection may aid the development of effective intervention strategies. Below, we elaborate on two other examples of recent important zoonotic events that led to sustained virus transmission in the human host, and the role that genomics has played in the elucidation of their pathogenesis thus far.

## Influenza Virus

Influenza is caused by RNA viruses of the Orthomyxoviridae family. Whereas fever and coughs are the most frequent symptoms, in more serious cases a fatal pneumonia can develop, particularly in the young and the elderly. Typically, influenza is transmitted through the air by coughs or sneezes, creating aerosols containing the virus; but influenza can also be transmitted by bird droppings, saliva, feces, and blood. Birds and pigs play an important role in the emergence of new influenza viruses in humans. Fecal sampling of migratory birds has revealed that they harbor a large range of different subtypes of influenza A viruses [8]. Some wild duck species, particularly mallards, are potential long-distance vectors of highly pathogenic avian influenza virus (H5N1), whereas others, including diving ducks, are more likely to act as “sentinel” species that die upon infection [9]. Following the introduction of a new pandemic influenza A virus subtype from an avian reservoir, either directly or via another mammalian species such as the pig, the virus may continue to circulate in humans in subsequent years as a seasonal influenza virus. In the past century, three major influenza epidemics resulted in the loss of many millions of lives. Spanish flu alone caused the deaths of more than 50 million people by the end of World War I in 1918. The 2009 outbreak of a new H1N1 virus (causing “swine flu”) that started in Mexico further illustrates the pandemic potential of influenza A viruses.

**Figure 1:**
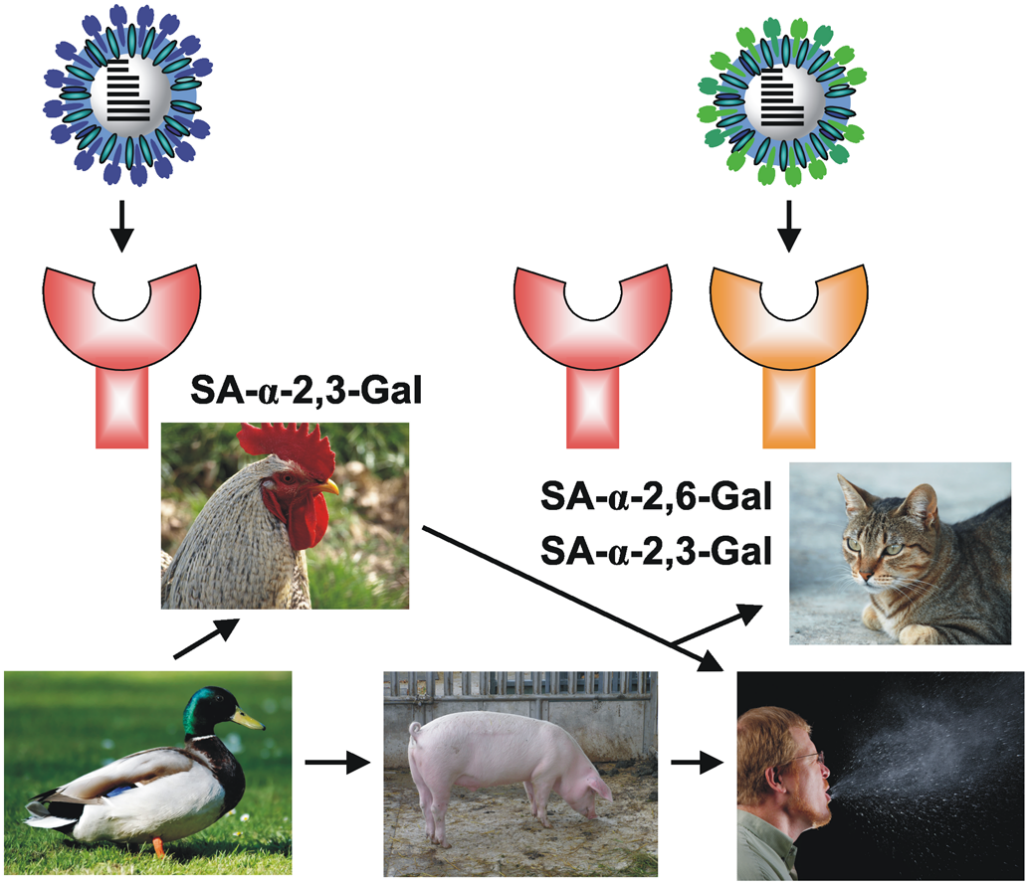
Zoonotic transmission of influenza A virus. The hemagglutinin of avian influenza A viruses (blue) preferentially bind to oligosaccharides that terminate in sialic acid–α-2,3-Gal (red), whereas the hemagglutinin on human influenza A viruses (green) prefer oligosaccharides that terminate in sialic acid–α-2,6-Gal (orange). Fatal viral pneumonia in humans infected with the H5N1 subtype of avian influenza A viruses is likely due to the ability of these viruses to attach to and replicate in the lower respiratory tract cells, which have sialic acid-α-2,3-Gal terminated saccharides. The horizontal arrows indicate interspecies transmission, including the transmission from an avian or porcine reservoir into the human species. Image credit: Bart Haagmans, Erasmus MC. Original images (left to right, from top to bottom) by Roman Köhler, Alvesgaspar, Anton Holmquist, Joshua Lutz, and CDC.

After introduction of a new influenza A virus from an avian or porcine reservoir into the human species, viral genomics studies are essential to identify critical mutations that enable the circulating virus to spread efficiently, interact with different receptors, and cause disease in the new host. For example, the importance of residue 627 of the PB2 protein of the viral polymerase in determining species restriction has been demonstrated through these kinds of approaches [10]. Furthermore, changes in the hemagglutinin molecules may allow influenza A viruses to switch receptor specificity. The hemagglutinin of avian H5N1 influenza viruses preferentially binds to oligosaccharides that terminate with a sialic acid–α-2,3-Gal disaccharide, whereas the hemagglutinins of mammalian influenza A viruses prefer oligosaccharides that terminate with sialic acid–α-2,6-Gal (Figure 1). Fatal viral pneumonia in humans infected with avian H5N1 viruses is partly due to the ability of these viruses to attach to and replicate in the cells of the lower respiratory tract, which have oligosaccharides that terminate in sialic acid–α-2,3-Gal disaccharide [11],[12]. The sequence of the hemagglutinin protein may also affect its binding affinity for neutralizing antibodies. Understanding the relationship between genetic diversity and antigenic properties of these viruses [13] may help to predict the emergence of influenza viruses and to develop effective vaccines.

Microarray-assisted mRNA expression profiling of emerging zoonotic viral infections, including influenza A virus, is used to phenotype the host response in great detail. By comparing mRNA expression in individuals infected with an emerging virus to expression in individuals infected with a related established virus, researchers can generate a “molecular fingerprint” of the host response genes or pathways specifically involved in the often-exuberant host responses to the emerging virus. By using genetically engineered influenza A viruses, a role for the nonstructural NS1 viral protein in evasion of the innate host response has been demonstrated [14]. Interestingly, the NS1 protein derived from the 1918 Spanish H1N1 pandemic influenza virus blocked expression of interferon-regulated genes more efficiently than did the NS1 protein from established seasonal influenza viruses [14]. Other genomics studies of genetically engineered influenza A viruses containing some or all of the gene segments from either the 1918 H1N1 virus or the highly pathogenic avian influenza A virus (H5N1), suggest that these highly pathogenic influenza viruses induce severe disease in mice and macaques through aberrant and persistent activation of proinflammatory cytokine and chemokine responses [15]–[18]. Application of genomics tools not only supports the elucidation of mechanisms underlying pathogenesis but may also help to identify leads for therapeutic intervention. In ferrets, H5N1 infection induced severe disease that was associated with strong expression of interferon response genes including the interferon-γ-induced cytokine CXCL10. Treatment of H5N1-infected ferrets with an antagonist of the CXCL10 receptor (CXCR3) reduced the severity of the flu symptoms and the viral titers compared to the controls [19], clearly demonstrating the potential of biological response modifiers for the clinical management of viral infections. The host evasion and evolution of influenza virus is further discussed in [20].

## SARS-CoV

Coronaviruses (CoVs) primarily infect the upper respiratory and gastrointestinal tract of mammals and birds. Five different currently known CoVs infect humans and are believed to cause a significant percentage of all common colds in human adults. Surprisingly, recent studies revealed that approximately 6% of bats sampled in China were positive for CoVs [21]. Subsequent phylogenetic studies revealed that bat CoVs that resembled human SARS-CoV clustered in a putative group comprising one subgroup of bat CoVs and another of SARS-CoVs from humans and other mammalian hosts. According to the current hypothesis SARS-CoV has arisen by recombination between two bat viruses. Phylogenetic analysis of SARS-CoV isolates from animals indicate that the resulting bat virus was transmitted first to palm civets (*Paguma larvata*), a wild cat-like animal hunted for its meat, and subsequently to humans at live animal markets in southern China [22].

**Figure 2:**
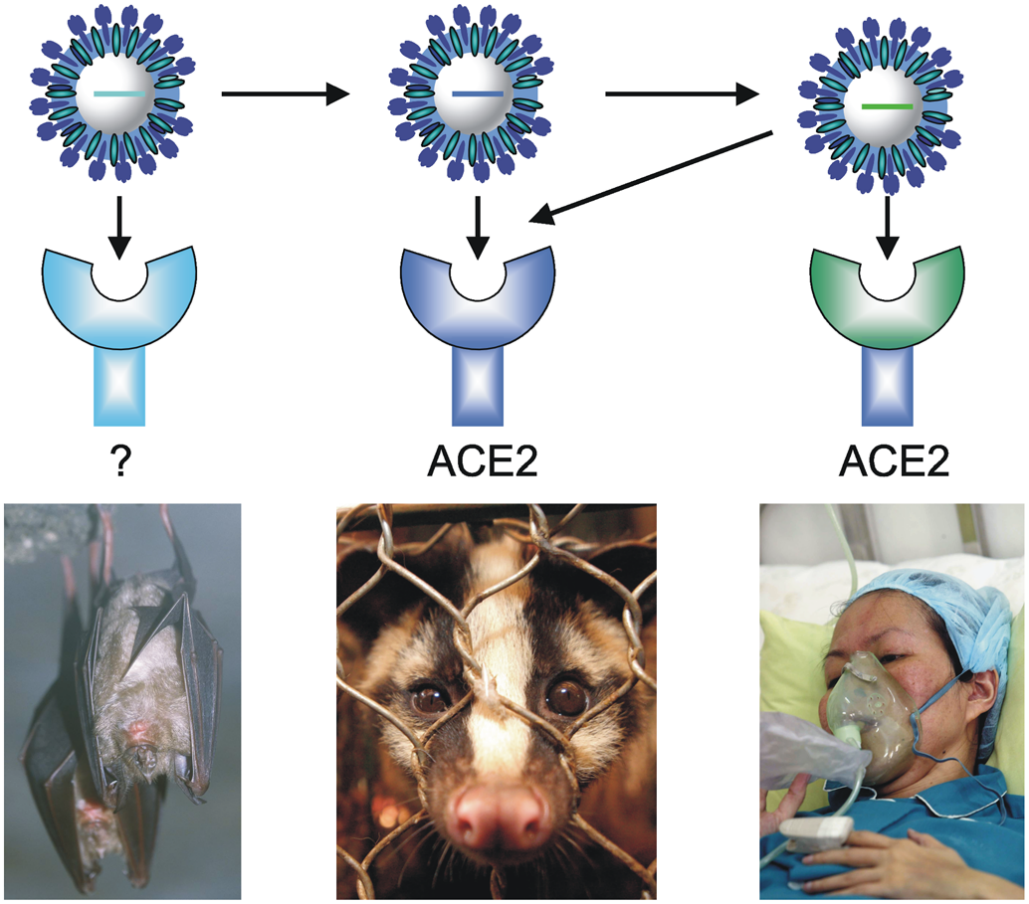
Zoonotic transmission of SARS-CoV. Genomic analyses provided evidence that genetic changes in the spike gene of SARS-CoV from bats (left) and civet cats (center) are essential for the animal-to-human transmission (horizontal arrows). Species-to-species genetic variation in the (thus far unidentified) viral receptor in bats and in the *angiotensin converting enzyme 2 (ACE2)* gene, encoding the SARS-CoV receptor in civet cats and humans also affects the efficiency with which the virus can enter cells (vertical arrows). The SARS-CoV that caused the epidemic evolved a high affinity for both civet (center) and human (right) ACE2 receptors (indicated by the single diagonal and the right side vertical arrow). Image credit: Bart Haagmans, Erasmus MC. Original images (left to right) by Dodoni, Paul Hilton, and Hoang Dinh Nam.

Genome analyses have provided evidence that genetic variation in the spike gene of these viruses from civets is associated with increased transmission of the virus [21]. In addition, species-to-species variation in the sequence of the gene *angiotensin-converting enzyme 2 (ACE2)*, which encodes the SARS-CoV receptor, also affects the efficiency by which the virus can enter cells [23]. By a combination of phylogenetic and bioinformatics analyses, chimeric gene design, and reverse genetics–aided generation of viruses that encode spike proteins of diverse isolates, researchers have reconstructed the events that led to the emergence of a virus able to spread efficiently in humans [24]. Structural modeling predicted that the SARS-CoV that caused the epidemic had an increased affinity for both civet and human ACE2 receptors due to adaptation (Figure 2). Subsequent functional genomics studies of these viruses in diverse species provided further insight into the role of specific host genes involved in the pathogenic response [25],[26]. The pathological changes observed in the lungs are initiated by a disproportionate innate immune response, illustrated by elevated levels of inflammatory cytokines and chemokines, such as CXCL10 (IP-10), CCL2 (MCP-1), interleukin (IL)-6, IL-8, IL-12, IL-1β, and interferon-γ [27]. These clinical data were confirmed experimentally by demonstrating that SARS-CoV infection of diverse cell types induces a range of cytokines and chemokines, thus providing a conceptual framework for SARS-CoV pathogenesis. Host genome expression analyses of various animal hosts and humans with different outcomes of infection indicated differential activation of innate immune genes in, for example, aged subjects compared to young subjects. Importantly, treatment of aged macaques with pegylated interferon-α (i.e. interferon-α covalently modified with polyethylene glycol polymer chains, to enhance its bioavailability) reduced SARS-CoV replication and pathogenic responses [28]. Thus, host genomics analysis may provide markers of pathogenesis and leads for therapeutic intervention, as in this example of SARS-CoV infection.

## Challenges for the Future

Rapid identification of newly emerging viruses through the use of genomics tools is one of the major challenges for the near future. In addition, the identification of critical mutations that enable viruses to spread efficiently, interact with different receptors, and cause disease in diverse hosts through, for instance, enhanced viral replication or circumvention of the innate and adaptive immune responses, needs to be further expanded. Although microarray-assisted transcriptional profiling can provide us with a wealth of information regarding host genes and gene-interacting networks in virus–host interactions, future research should focus on combining data obtained in different experimental settings. Therefore, the careful design of complementary sets of experiments using different formats of virus–host interactions is absolutely needed for successful genomics studies [29]. Special attention should be addressed to the comparative analysis of the host response in diverse animal species. Thus far a limited number of laboratory animal species has been studied, but the recent elucidation of the genome of several other animal species will provide tools to decipher the virus–host interactions in the more relevant natural host. Recent developments in the sequencing of the RNA transcriptome may aid this development. Ultimately, microarray technology may also extend to genotyping of the human host by SNP analysis, to identify markers of host susceptibility and severity of disease, that can be used in tailor-made clinical management of disease caused by emerging infections. Comparative analysis of host responses to emerging viruses may also point toward a similar dysregulated host response to a range of emerging virus infections, enabling the rational design of multipotent biological response modifiers to combat a variety of emerging viral infections. By focusing on broad-acting intervention strategies rather than on the discovery of a newly emerging pathogen that is not characterized yet, we may be able to protect ourselves from several unexpectedly emerging infections with the same clinical manifestations. This approach may readily reduce the burden of disease and time will be gained to design preventive pathogen specific intervention strategies such as antiviral therapy or vaccination. Clearly, for all stages of combating emerging infections, from the early identification of the pathogen to the development and design of vaccines, application of sophisticated genomics tools is fundamental to success.
